# Biosimilars versus biological therapy in inflammatory bowel disease: challenges and targeting strategies using drug delivery systems

**DOI:** 10.1007/s10238-025-01558-6

**Published:** 2025-04-05

**Authors:** Ahmed Aljabri, Ghareb M. Soliman, Yasmin N. Ramadan, Mohammed A. Medhat, Helal F. Hetta

**Affiliations:** 1 Department of Pharmacy Practice, Faculty of Pharmacy, King Abdulaziz University, Jeddah, Saudi Arabia; 2 Department of Pharmacy Practice, Faculty of Pharmacy, University of Tabuk, Tabuk, 71491 Saudi Arabia; 3 Department of Pharmaceutics, Faculty of Pharmacy, University of Tabuk, Tabuk, 71491 Saudi Arabia; 4 Department of Microbiology and Immunology, Faculty of Pharmacy, Assiut University, Assiut, 71515 Egypt; 5 Department of Tropical Medicine and Gastroenterology, Faculty of Medicine, Assiut University, Assiut, 71515 Egypt; 6 Division of Microbiology, Immunology and Biotechnology, Department of Natural Products and Alternative Medicine, Faculty of Pharmacy, University of Tabuk, Tabuk, 71491 Saudi Arabia

**Keywords:** Inflammatory bowel disease, Biologics, Infliximab, Adalimumab, Ustekinumab, Biosimilars, Drug delivery system

## Abstract

Inflammatory bowel disease (IBD) is a multifactorial illness with a climbing prevalence worldwide. While biologics are commonly prescribed especially for severe cases, they may worsen patients’ outcomes due to financial burden. Consequently, there has been an increased focus on biosimilars to improve overall disease outcomes by maintaining similar efficacy and safety while minimizing the cost of therapy. Infliximab-dyyb was the first biosimilar approved by US-FDA for IBD. Since that, the US-FDA approved 14 biosimilars with different mechanisms of action and different routes of administration for IBD patients (four infliximab biosimilars, nine adalimumab biosimilars, and most recently one ustekinumab biosimilar). It should be noted that more biologics are in the pipeline as golimumab and natalizumab patents are set to expire in the near future, and biosimilars are now in pre-clinical to phase 3 trials. Different studies have evaluated biologics' effectiveness and safety and concluded that the majority of available biosimilars are efficacious and have similar adverse effect profiles compared to their reference biologics. It is worth mentioningthat post-marketing surveillance reports revealed some risks associated with biosimilars which should be taken into consideration in future research and clinical trials to avoid health hazards. Most biologics and biosimilars are administered parenterally which results in several drawbacks such as raised risk of infections, hypersensitivity, autoimmunity, development of malignancies, liver toxicity as well as worsening of heart failure. Several drug delivery systems based on passive and active targeting mechanisms are under active investigation to overcome these limitations. This review sheds light on the emergence of biologics and biosimilars as alternatives in IBD management, the differences between them, challenges and risks, and future perspectives in IBD therapy and new trends in drug delivery systems.

**Graphical Abstract:**

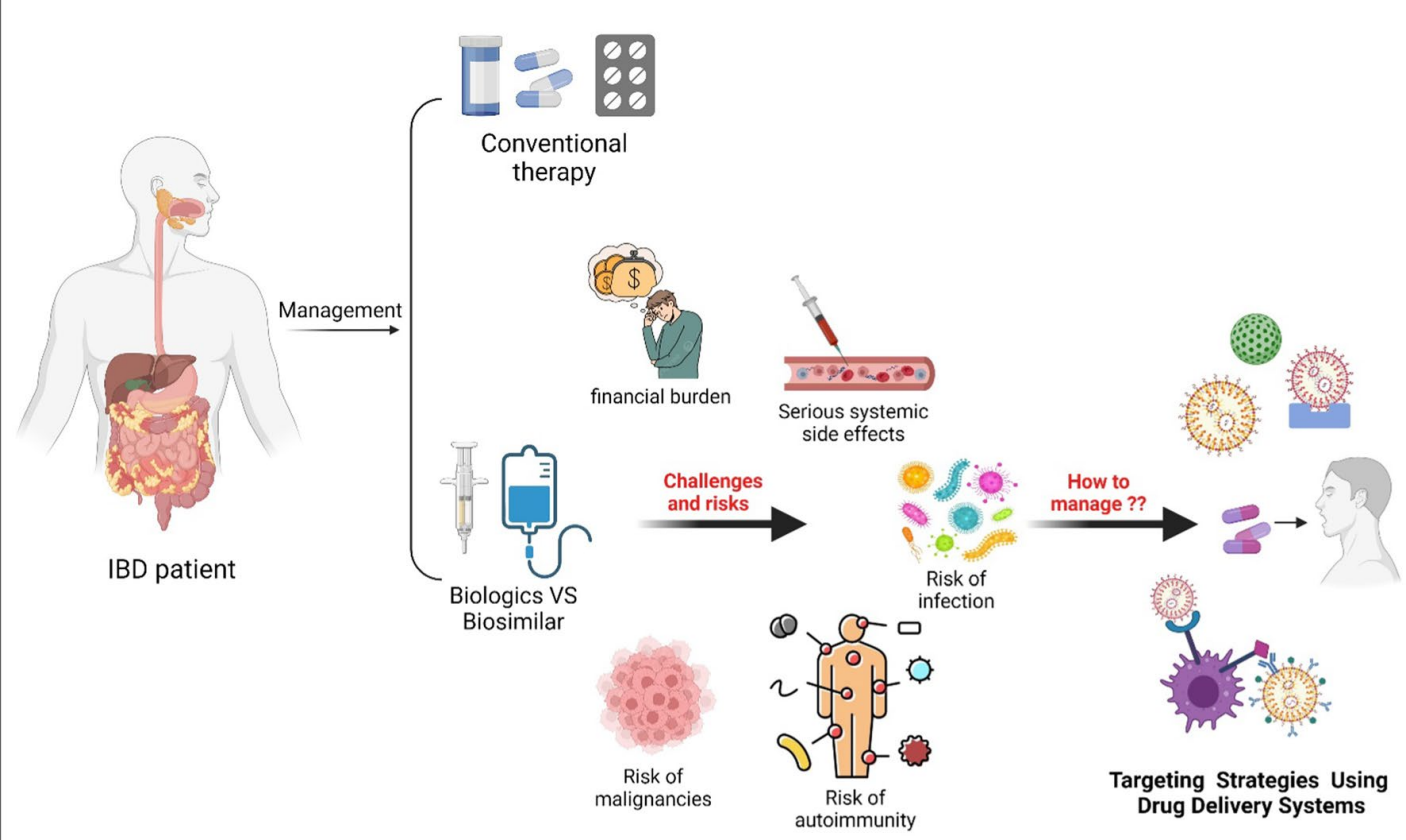

## Introduction

Inflammatory bowel disease (IBD) is a chronic, relapsing inflammatory condition of the gastrointestinal tract (GIT), driven by a complex interplay of genetic predisposition, environmental influences, microbial dysbiosis, and dietary factors [1–6]. It primarily encompasses two major forms: ulcerative colitis (UC), which predominantly affects the colon and rectum, and Crohn’s disease (CD), which can involve any segment of the GIT, often presenting with transmural inflammation and a patchy distribution [2, 7–14]. Both conditions lead to severe symptoms, including abdominal pain, diarrhea, and malnutrition, significantly impacting patients' quality of life and posing substantial healthcare challenges [1, 15].

The management of IBD is tailored to disease severity and typically involves a step-up approach, ranging from conventional therapies like aminosalicylates and corticosteroids for mild cases to biologics or biosimilars for moderate to severe forms [16, 17]. Biologics have revolutionized IBD treatment by offering targeted mechanisms of action. However, their high cost has limited accessibility, particularly in low- and middle-income countries, contributing to significant disparities in care.The rising global prevalence of IBD, projected to increase 4–6 fold by 2030, has resulted in a growing demand for biologics, imposing a substantial financial burden on healthcare systems [18–21].

To address these financial barriers, biosimilars—biological products highly similar to approved reference biologics—have been introduced as affordable alternatives. Over the past decade, several biosimilars have been approved for IBD, demonstrating comparable safety, efficacy, and immunogenicity profiles [21–29]. While biosimilars are considered safe and effective, real-world data reveal some risks, including adverse drug reactions identified through post-marketing surveillance [30]. These concerns underscore the need for advancements in drug delivery systems (DDSs) to enhance the safety and effectiveness of biologics and biosimilars..

Drug delivery systems (DDSs) offer promising solutions to address limitations in biologics and biosimilars, such as poor stability, immunogenicity, and the need for targeted delivery. Advanced DDSs can protect therapeutic molecules from enzymatic degradation, enhance bioavilability, and facilitate targeted delivery to inflamed intestinal tissues while minimizing systemic side effects [31]. These systems also enable controlled drug release, ensuring sustained therapeutic levels, reducing immunogenic responses, and improving treatment adherence. Innovations such as nanoparticles, hydrogels, and oral delivery platforms are being explored to optimize the stability and efficacy of biologics and biosimilars in IBD management [32, 33].

This review delves into the evolving role of biologics and biosimilars in IBD treatment, emphasizing their adverse effects and the potential of DDS technologies to address these challenges. By integrating these advancements, we aim to provide insights into enhancing therapeutic outcomes and improving the quality of care for IBD patients worldwide.

## General overview of the pathogenesis of IBD

IBD is a multifactorial complex inflammatory disease. Full comprehension of IBD pathogenesis is still out of reach. Increased incidence in specific populations across the world shows a strong hereditary component to the development of IBD. The genomic study of IBD patients, particularly through genome-wide association studies (GWAS), has uncovered many pathways implicated in IBD pathogenesis [34–36]. According to GWAS, IBD is a polygenic disorder triggered by several genetic polymorphisms [35]. A meta-analysis of GWAS data reported 240 loci linked to IBD [36–38]. However, the current epidemiologic trends also show that environmental variables are important in the pathophysiology of UC and CD. This mostly results from the industrialization of developing countries [5, 39]. Furthermore, most environmental factors may promote IBD pathogenesis by affecting the gut microbiome [40]. However, gut microbiome alteration triggers intestinal inflammation only in the presence of disrupted intestinal barrier integrity. Lastly, immune system activation is dependent on the degree of balance between effector (Th) and regulatory (Treg) cells present in the intestinal mucosa, which has also been found to be dysregulated in IBD patients [2, 41].

**Fig. 1:**
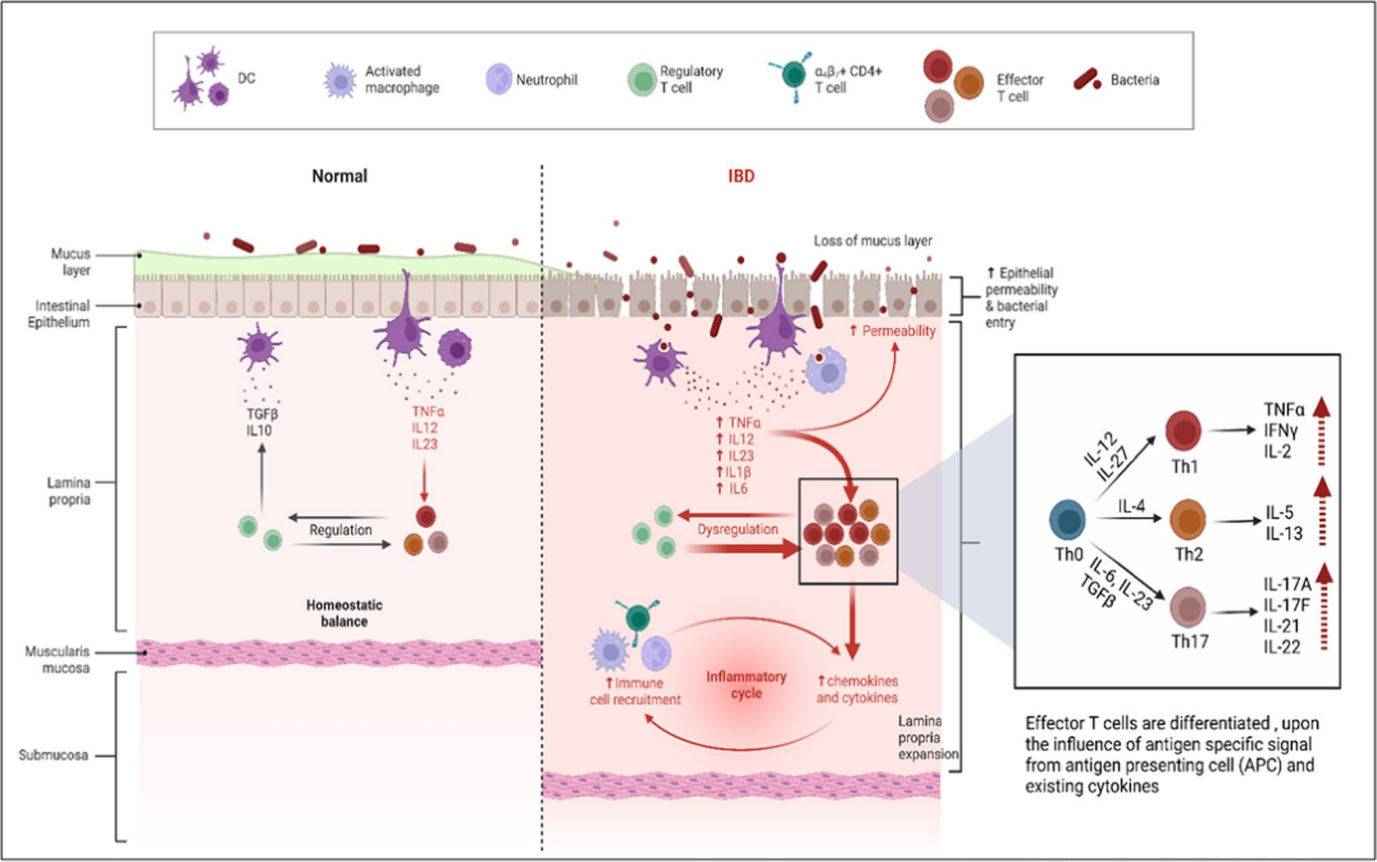
Homeostatic balance and pathogenesis in IBD. In normal healthy conditions, the gut microbiome maintains homeostatic balance with a proportional number of pro and anti-inflammatory cytokines. In IBD, exposure to environmental factors leads to gut microbiome dysbiosis activation of immune response and inflammatory cascade through the destruction of mucus layer and tight junctions. Uncontrolled activation of immune response leads to activation of effector T-cells with secretion of different types of inflammatory cytokines and progression of inflammation in the gut wall. Created with BioRender

Altogether, IBD pathophysiology involves the interplay of genetic susceptibility and environmental effects on the gut microbiome that triggers immune activation through a damaged intestinal barrier (Fig. 1).

## US-FDA-approved and pipeline biologics and small molecules for IBD

The management of IBD has undergone a significant development since the advent of biological therapy which inhibits proinflammatory cytokines and leukocyte migration, and mechanisms thought to be key players in the pathophysiology of both UC and CD. They can block the chemical massages from the immune system that trigger inflammatory cascades [42].

**Fig. 2:**
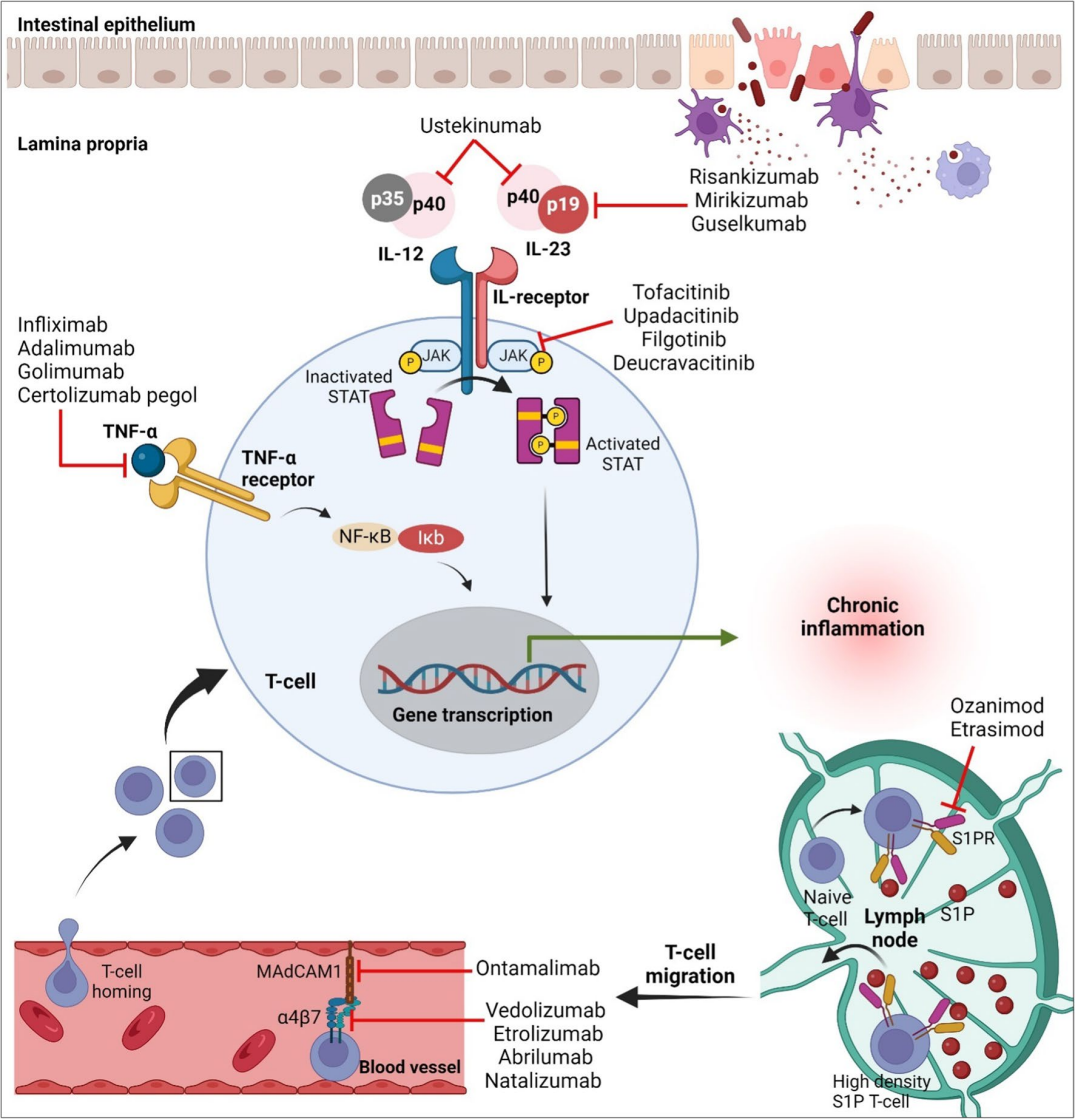
Therapeutic targets of biologics and small molecules indicated for IBD management. Biologics and small molecules help in managing IBD by interfering with inflammatory cascades by: inhibiting lymphocyte trafficking and reducing inflammation by modulating Sphingosine-1-phosphate receptor** (**S1PR), Inhibiting inflammatory cell recruitment by blocking α4β7 integrin or MAdCAM receptor, blocking several pro-inflammatory cytokines such as TNF-α, IL-12 or/and IL-23, or inhibiting many cytokine pathways through blocking Janus kinase (JAK) signaling pathway. Created with BioRender

Several targets for biologics and small molecules are available to treat IBD (Fig. 2), which will be discussed in the following sections.

**Tumor necrosis factor-alpha (TNF-α) blockers** were the first class of biologics authorized for the treatment of IBD in the US [43–45]. TNF-α is a proinflammatory cytokine that plays a critical role in several inflammatory cascades. So, blocking and neutralizing this proinflammatory cytokine pauses inflammatory reactions and the resulting tissue damage and helps heal the intestine [46]. Infliximab (Remicade®) was the first anti-TNF-α, chimeric IgG1 monoclonal antibody (mAb), approved for IBD in 1998. Following that, other anti-TNF-α were approved such as adalimumab (Humira®) and golimumab (Simponi®), a fully human anti-TNF-α mAb. Adalimumab was approved in 2002 for UC and CD patients, whereas golimumab was approved in 2009 for moderate to severe UC patients with failed conventional treatment [47–49]. Certolizumab pegol (Cimzia®) is another anti-TNFα mAb that was approved in 2008 for CD [50].

**Integrin blockers** inhibit white blood cells (leukocytes) from entering the GIT and causing inflammation. Integrin is a glycoprotein receptor on the cell surface that guides homing of leukocytes to inflamed tissue. In GIT, α4β7 integrin binds to mucosal addressin cell adhesion molecule (MAdCAM). IBD patients have elevated MAdCAM1 expression and α4β7 positive cell infiltration [51]. Ontamalimab, a fully human IgG2 mAb against MAdCAM, was developed as a therapy for moderate-to-severe UC and CD. Vermeire et al. [52] demonstrated that Ontamalimab 75 mg was efficacious and safe for induction and maintenance therapy for moderate-to-severe UC patients. Natalizumab (Tysabri®) is the first integrin blocker, humanized IgG4 mAb against α4 chain, that was approved in 2004 for CD. This mAb is not specific for α4β7 that is present in the gut [53, 54]. So, a specific α4β7 integrin blocker, vedolizumab, was developed and approved in 2014 [55]. Vedolizumab (Entyvio®) is a humanized IgG1 mAb that functions by blocking the binding of α4β7 integrin to MAdCAM-1 with subsequent inhibition of leukocytes homing to intestinal tissue and reduction in intestinal inflammation. Vedolizumab is beneficial for inducing and maintaining remission in UC and CD patients. Furthermore, it has been approved for use in adult patients with moderate-to-severe UC and CD and does not respond well to anti-TNF-α or conventional treatment [56–59]. Approval for use in pediatric IBD patients is delayed, despite promising preliminary findings from clinical trials [60, 61]. Other integrin blockers, abrilumab [62, 63], show promising results in phase 2b clinical trials and wait for approval from regulatory agencies.

**Interleukin blockers** target IL-12 and/or IL-23, two proteins implicated in GIT inflammation. IL-23 is composed of two subunits: p40 and p19, whereas IL-12 is composed of p40 and p35. It seems that the p40 subunit is common between IL-12 and 23 [64]. Ustekinumab (Stelara®) is a fully human IgG1 mAb that targets the p40 subunit. Thus, intestinal inflammation is prevented by ustekinumab by preventing the binding of IL-23 and IL-12 to the IL-12 receptor on inflammatory cells. Ustekinumab is indicated for moderate to severe UC and CD patients [65, 66].

Given that IL-12 and IL-23 mediate different inflammatory responses, targeting the p19 subunit specifically is a new approach to deactivating the IL-23 pathway. Selective suppression of IL-23 impedes Th17 development and IL-17 overexpression, as well as the recruitment of inflammatory cells such as macrophages, dendritic cells, and neutrophils, which in turn reduces intestinal inflammation [67, 68]. For example, risankizumab (Skyrizi®) is a fully human IgG mAb that selectively inhibits the p19 subunit in IL-23. Risankizumab was approved in 2022 for CD and recently for UC [69], and clinical trials demonstrated its efficacy and safety [70–72]. Risankizumab is effective for both naïve patients and those previously exposed to biologics. However, it was more effective in patients who failed only one biologic [72]. Mirikizumab (Omvoh®) is the first selective p19 mAb for managing moderate to severe UC in adults who have not responded to conventional or biologic therapy [73, 74]. Guselkumab is another selective IL-23 (p19) mAb, was approved for psoriasis, and now shows a promising effect for CD [75] and UC [76] patients but is still waiting for approval.

**Janus kinase (JAK) inhibitors** influence many cytokine pathways and reduce inflammation. JAK is an intracellular tyrosine kinase (TYK). JAK family consists of JAK1, JAK2, JAK3, and TYK2. JAKs are a crucial component of the JAK/STAT signaling system, which controls a variety of intracellular processes. JAK3 expression is limited to hematopoietic cells, however, JAK1, JAK2, and TYK2 are found in various tissues [77].

JAK inhibitors can suppress several cytokine pathways associated with inflammatory disorders, including IL-2, IL-4, IL-7, IL-9, IL-15, and IL-21. They are the first oral therapy for IBD treatment. They target many cytokine pathways, unlike monoclonal antibodies, which only block one pathway. Additionally, they operate quickly, which leads to a quick improvement and remission of symptoms [78, 79].

Tofacitinib (Xeljanz®) is the first JAK inhibitor approved for induction and maintenance treatment of moderate to severe UC patients. Tofacitinib is a nonselective JAK inhibitor that targets both JAK1 and JAK3. Unfortunately, previous ORAL surveillance trials found a link between tofacitinib and systemic adverse effects in rheumatoid arthritis patients, including cardiovascular events, venous thromboembolism, and malignancies [80]. Therefore, more specific JAK inhibitors are required for IBD.

Upadacitinib (Rinvoq®) is a selective JAK1 inhibitor and has been approved for use in UC [81] and CD [82] patients. In a recent study, upadacitinib was reported to possess a promising effect on induction and maintenance therapy for difficult-to-treat UC patients (loss of response, previous inadequate response, or intolerance to previous treatments including biologics) [83]. Additionally, a recent post-hoc investigation indicated that upadacitinib had a quick beginning of activity on UC symptoms starting from day 1 of the induction therapy [84]. Upadacitinib's long-term safety study is anticipated to be finished in 2027 (https://clinicaltrials.gov/ identifier NCT03006068).

Filgotinib, a specific JAK1 inhibitor, has shown promising efficacy in inducing remission in CD patients. A phase II clinical trial reported that filgotinib can induce remission and healing in CD patients. Unfortunately, improved patients experienced serious side effects and serious infections than placebo [85]. Additionally, in rheumatoid arthritis clinical trials, filgotinib results in testicular toxicity. So, US-FDA does not approve the use of filgotinib due to its toxicity [86].

Overall, these novel selective JAK inhibitors provide excellent potential for IBD therapy, but their safety profiles should not be neglected.

The highly selective TYK2 inhibitor deucravacitinib, which has no effect on JAK3, is currently undergoing clinical trials in patients with IBD. This drug may provide a novel treatment option for these patients [87].

**Sphingosine-1-phosphate receptor (S1PR) modulator** that inhibits lymphocyte trafficking and reduces inflammation. Sphingosine-1-Phosphate (S1P) is a lipid molecule that possesses regulatory roles in the immune system. It attaches to certain receptors and activates them [88].

Ozanimod (Zeposia®) is a selective S1PR modulator that binds to S1PR-1 and S1PR-5, inhibiting the migration of lymphocytes from lymph nodes with subsequent reduction in the number of circulating lymphocytes at the site of inflammation. Ozanimod was approved as an induction and maintenance therapy for moderately to severely active UC patients [89]. Ozanimod has a potential efficacy in CD; phase 3 findings will be announced in 2024 for maintenance and in 2026 for open-label trial [90, 91].

Etrasimod (Velsipity®) is the second oral S1PR-1, S1PR-4, and S1PR-5 modulator [92, 93]. Etrasimod was recently approved by the US-FDA, in October 2023, for moderate to severe UC [94].

Biologics were launched onto the market in 1998, and their patents are about to expire. Annual direct expenditures for IBD can reach up to $41,000 per patient, and medications account for almost 50% [95]. The prescription of these biologics is a major contributor to this financial issue. The economic burden is worsened and complicated by insurance companies altering their rules on covering these biologics. Data from 2007 to 2015 revealed that the percentage of patients receiving biologics increased from 5 to 16% in UC and nearly doubled in CD, from 20 to 40% [96]. Considering the serious financial burden on the government and taxpayers caused by IBD treatment, it is logical to focus on solutions that can reduce costs while providing the same level of healthcare. These situations, together with commercial pressure connected to the expiring patents of biologics, push the manufacturers to develop a comparable copy of these biologics, known as "biosimilars."

## Biologics versus biosimilars

Biologics are large and complex proteins derived from live organisms, which can include animal cells and microorganisms like yeast and bacteria, through highly complex manufacturing processes. Biologic medications include gene therapies, stem cell therapies, recombinant proteins, as well as monoclonal antibodies (mAbs). However, those approved for IBD are mAbs. They are frequently utilized to treat a wide range of serious and fatal life-threatening illnesses [97, 98]. Biologics differ from conventional medicines in terms of material sources, structural complexity, manufacturing method, and regulatory requirements [97]. Therefore, the cost of biologics may occasionally be greater, which may prevent certain patients from accessing them [98].

Unlike generic drugs, which have identical active components, biosimilars are comparable but not identical to their originators. According to the US-FDA, a biosimilar is a biological product that is potent, pure, and safe and that is "highly similar to and has no clinically meaningful differences from an existing US-FDA-approved reference product". In other words, biosimilars are equivalent to the reference biologics regarding safety and efficacy [98, 99]. Biosimilars were developed to broaden access to biologics and hence enhance patient outcomes [99]. In some situations, pharmaceutical companies may look for an extra US-FDA designation for a biosimilar drug so that it can be considered interchangeable with reference biologics. Although this extra designation is not necessary for biosimilars, several pharmaceutical companies ask for it in order to facilitate the switching of a biosimilar with reference biologics, according to state laws [99].

All proteins (reference/originators and biosimilars) can undergo a variety of post-translational modifications (PTMs) as a result of the utilization of eukaryotic cellular systems in their manufacture. Variability in PTMs can occur not just between biosimilars and reference biologics, but also between various batches of reference biologics [100]. So, it is typical and anticipated for biosimilars and reference biologics to change somewhat between batches of the same drug. This means that biologics cannot be precisely copied, which explains why biosimilars differ from the reference biologics [99]. However, biosimilars must have no clinically significant changes from their reference biologics. On top of that, biosimilars must be administered in the same manner (same mode of administration), in the same potency and dosage form, and with the same potential adverse effects [99].

Regarding the regulatory path, all medications are subjected to extensive testing before being approved [101]. Compared to biologics, the approval procedure for biosimilars is more streamlined. The data submitted during the biologic approval process is intended to prove its safety and effectiveness, while the data presented during the biosimilar approval process is intended to demonstrate the clinical equivalence between it and the reference biologic [102, 103].

Lastly, regarding the cost, biosimilars could be less costly than biologics, which partly explains their streamlined development process. The Association for Accessible Medicines states that the competition between biosimilars not only reduces the cost of biosimilars but also references biologics, saving $7.9 billion in 2020 and more than $12.6 billion over the previous ten years [104]. The main differences between biologics and biosimilars are listed in Table 1**.**

**Table 1:** Main differences between biologics and biosimilars

	Biologics	Biosimilars
Production process	Biologics are large and complex proteins derived from live organisms, which can include animal cells and microorganisms like yeast and bacteria, through highly complex manufacturing processes	Like biologics, produced through intricate, multistep complex processes using live organisms
Complexity	More complex	Less complex
Regulation	Must pass rigorous regulatory approval procedures and require submission of biologics license application (BLA)	The approval procedure is more streamlined and requires the submission of an abbreviated biologics license application (aBLA)
Patent duration	Up to 20 years	No patent licensing
Interchangeability	Not applicable	Some biosimilars may be regarded as interchangeable with the reference biologic
Development costs	Approximately $2 billion	Approximately $100–250 million
Indication extrapolation	Not permitted	Case-by-case basis

## The emergence of biosimilars as an alternative to biologics

Biosimilars are biotherapeutic agents that are comparable to reference products that have been authorized and licensed by the US-FDA for several inflammatory disorders including IBD. Biosimilars provide greater access to life-saving drugs at possibly reduced costs. In terms of safety, purity, potency, and efficacy, biosimilars are identical to their respective reference biologics. The mechanism of action of biosimilars is also comparable to their reference product. However, they only differ in some inactive ingredients [22, 105]. To date, 45 biosimilars have been approved by the US-FDA, with the most recent approval scheduled in December 2023 [23].

For US-FDA approval, manufacturers must prove a biosimilarity between the biosimilar and reference product with respect to structural and functional aspects, immunogenicity, and bioactivity [106]. Following US-FDA approval for the biosimilar based on medical equivalence in one disease condition, it may be authorized for use in other conditions approved for the reference product without further research or analysis. This approach, called extrapolation, aims to speed up the approval process so that patients can receive treatment more easily and at lower costs [107]. Moreover, in terms of interchangeability, switching between reference biologics and biosimilars needs further approval from the FDA and shouldn’t decrease effectiveness or increase the risks of treatment [106].

Several biosimilars are now licensed for managing IBD. To date, the US-FDA approved four infliximab biosimilars, nine adalimumab biosimilars, and most recently one ustekinumab biosimilar in the management of IBD [23].

### Infliximab biosimilars

Infliximab-dyyb (INFLECTRA® -Celltrion, or CT-P13), is the first infliximab biosimilar that was approved by US-FDA in April 2016 for IBD that was first used in certain countries such as South Korea and India [108].

Subsequently, the US-FDA approved infliximab-abda (RENFLEXIS®) [109] and infliximab-qbtx (IXIFI®) in May and December 2017, respectively [23]. Infliximab-qbtx is not available in the market as Pfizer company is the same manufacturer for infliximab-dyyb and decided to launch infliximab-dyyb instead [110]. Lastly, infliximab-axxq (AVSOLA®) was approved in December 2019 [23] (Table 1).

Infliximab biosimilars were first approved for IBD by extrapolation, but further studies have demonstrated their efficacy, safety, and comparability to reference infliximab in IBD [111]. In this regard, the NOR-SWITCH trial randomized 482 patients (about 51% with IBD) who were stable on long-term infliximab maintenance to either continue or switch to CT-P13. No apparent variation in the disease progression was observed in either group after 52 weeks of follow-up [112]. Following week 52, patients from this study participated in an open-label extension trial and may continue receiving CT-P13 or switch to CT-P13. After 26 weeks of follow-up, both groups showed no significant variations in disease worsening, supporting the effectiveness of the CT-P13 [113].

Most infliximab biosimilars are available as intravenous (IV) solutions. In October 2023, the US-FDA authorized the first and only subcutaneous formulation of infliximab-dyyb (Zymfentra®) [114]. Zymfentra® was indicated as maintenance therapy for moderate to severe UC and CD adult patients. This subcutaneous formulation is used for maintenance treatment, beginning at week 10 following IV infliximab induction and taken every two weeks at a dose of 120 mg. US-FDA approval was based on phase III biovital trial for UC (LIBERTY-UC) and CD (LIBERTY-CD) [115, 116].

The LIBERTY-UC trial is a randomized, placebo-controlled, double-blind study in which 438 patients, with moderate to severe active UC after IV induction of infliximab, were randomly allocated at week 10. At week 54, infliximab-dyyb had a substantially higher percentage of clinical remission (43.2%) than placebo (20.8%). The observed safety profile was similar in the infliximab-dyyb and placebo groups, according to the safety analysis results, with the most frequent side effects being COVID-19, injection site reaction, arthralgia, anemia, abdominal pain, and elevated alanine aminotransferase [115].

In the identically designed LIBERTY-CD trial, 343 patients with moderate to severe active CD following induction therapy were randomly allocated at week 10. At week 54, infliximab-dyyb had a higher clinical remission rate (62.3%) compared to placebo (32.1%). Similarly, the endoscopic response rate at week 54 was higher in the infliximab-dyyb (51.1%) compared to the placebo (17.9%). During the maintenance phase, the safety profile was generally comparable between the two studied groups. The most common side effects include COVID-19, injection site reaction, elevated alanine aminotransferase, upper respiratory tract infection, urinary tract infection, increased blood creatine phosphokinase, hypertension, headache, diarrhea, and leukopenia [116].

Additionally, a greater serum concentration of the drug was reported in a phase I trial after subcutaneous administration than in IV administration [117]. This result was subsequently supported in a phase II and III trial including rheumatoid arthritis patients [118]. Therefore, Zymfentra® is a "biobetter," a newer class of biologics designed to enhance efficacy, safety, and pharmacokinetic properties and reduce immunogenicity by increasing systemic drug concentration and stability [119].

Additional research demonstrating the safety and effectiveness of infliximab biosimilars has already been collected in previous reviews [20, 111]. Best practices for initiating and switching infliximab biosimilars have been found, demonstrating that using the correct tools may result in substantial cost reductions [120]. Infliximab biosimilars are more popular among gastroenterologists, with over 95% prescribing them in the last year (compared to 2016) [110].

In summary, the use of infliximab biosimilars is supported by all recently published data due to their comparable effectiveness and adverse effect profiles, which require more studies.

### Adalimumab biosimilars

In 2023, nearly 20 years after Humira's release, 9 adalimumab biosimilars were approved, causing a revolution in the adalimumab market [23] (Table 1). The US-FDA authorized the first adalimumab biosimilars in 2016; however, their commercial release was stalled until January 2023 owing to patent issues [121]. Since adalimumab is one of the most expensive biologics and brings in around $200 billion annually, the introduction of adalimumab biosimilars is expected to increase pricing competition and lead to cost reductions [122].

In VOLTAIRECD, a phase 3 randomized double-blind multicenter research, the safety, and efficacy of adalimumab-adbm were compared to the reference product in CD patients [123]. A total of 147 patients were enrolled in this study. At week 4, 90% of patients on adalimumab-adbm and 94% on reference adalimumab showed clinical responses. At week 24, respondents receiving adalimumab reference were transferred to adalimumab-adbm; 43% of those who persisted on the biosimilar and 45% of those who transferred experienced adverse events throughout weeks 24–56. The most prevalent side effects were injection site reaction and upper respiratory tract infections. Additional research supporting these findings evaluated several adalimumab biosimilars in patients with IBD and revealed no significant differences in biomarkers, adalimumab trough levels, or clinical remission between treatment-naïve and biosimilar-switch patients [124–132]. Injection site reactions were the most often reported side effects identified in these studies. In certain circumstances, patients showed or reported different responses to biosimilars than to the reference product. It's unclear if this is the result of a real response difference or the nocebo effect—a bad outcome brought on by the expectation that an intervention would be less effective or harmful. Nonetheless, every patient needs to be assessed separately and returning a patient to their favorite product [133].

Currently, the US-FDA has authorized the interchangeability designation for two adalimumab biosimilars: adalimumab-adbm (Cyltezo®, Boehringer Ingelheim) and adalimumab-afzb (Abrilada®, Pfizer) [110]. As an illustration, a patient may begin on Humira, shift to a biosimilar, return to Humira, and then shift again to the biosimilar. For adalimumab-adbm and adalimumab-afzb, interchangeability approval was provided based on VOLTAIRE-X and REFLECTIONS B538-12, respectively [134, 135]. Both trials were in phase III and included multiple switches between biosimilar and reference adalimumab. Comparable safety and efficacy were observed either in maintaining reference adalimumab or in multiple switching between reference and biosimilar.

Several differences were found between reference adalimumab and biosimilars, including available concentrations, inactive ingredients, and stability at room temperature [121, 136]. For example, Humira® is available in pen-injectors or prefilled syringes in low (40 mg/0.8 mL) and high (40 mg/0.4 mL) concentrations. The stability of this product lasts for 14 days at room temperature [136]. Additionally, Humira was produced in a formulation containing citrate, which causes pain and irritation upon injection. So, the majority of emergent biosimilars have been reformulated in citrate-free formulations to improve patient persistence and adherence to these pain-free formulations [137]. Humira® is available in a variety of injectable forms, including syringes, pens, and vials. Finally, Humira® is available in several concentrations (20,40, and 80 mg). In contrast, biosimilars are only available in 20 and 40 mg [138]. This will require increasing the number of injections in some cases to reach optimum concentration.

### Ustekinumab biosimilar

On October 31, 2023, the US-FDA authorized ustekinumab-auub, Wezlana®, as the first ustekinumab biosimilar with interchangeability designation. Wezlana, like reference ustekinumab (Stelara®), was approved to treat plaque psoriasis, and psoriatic arthritis, as well as moderate to severe UC and CD. Wezlana's US-FDA approval is supported by an extensive analysis of scientific data showing that it is very comparable to Stelara and that there are no clinically significant variations between the two medications in terms of potency, safety, or purity [139]. The indication for IBD was provided by extrapolation after a phase 3 trial in plaque psoriasis evaluated safety and effectiveness compared to the reference product. However, because of the terms of the settlement made by the manufacturer of the.

reference ustekinumab, this biosimilar medication will not be accessible until 2025 [140].

Regarding other biologics, golimumab, and natalizumab patents are set to expire in the near future, and biosimilars are now in pre-clinical to phase 3 trials [140] (Table 2).

**Table 2:** US-FDA-approved biosimilars indicated in IBD

Reference biologics	Biosimilar	Manufacturer	US-FDA approval date	Adult indication	Pediatric indication
Infliximab (Remicade®)	Inflectra® (infliximab-dyyb)	Celltrion	April 2016	UC and CD	UC and CD
	Renflexis® (infliximab-abda)	Samsung Bioepis	May 2017	UC and CD	UC and CD
	Ixifi® (infliximab-qbtx)	Pfizer	December 2017	UC and CD	UC and CD
	Avsola® (infliximab-axxq)	Amgen	December 2019	UC and CD	UC and CD
Adalimumab (Humira®)	Amjevita® (adalimumab-atto)	Amgen	September 2016	UC and CD	CD
	Cyltezo® (adalimumab-adbm)	Boehringer Ingelheim	August 2017	UC and CD	CD
	Hyrimoz® (adalimumab-adaz)	Sandoz	October 2018	UC and CD	CD
	Hadlima® (adalimumab-bwwd)	Samsung Bioepis	July 2019	UC and CD	None
	Abrilada® (adalimumab-afzb)	Pfizer	November 2019	UC and CD	None
	Hulio® (adalimumab-fkjp)	Mylan	July 2020	UC and CD	None
	Yusimry® (adalimumab-aqvh)	Coherus BioSciences	December 2021	UC and CD	CD
	Idacio® (adalimumab-aacf)	Fresenius Kabi	December 2022	UC and CD	CD
	Yuflyma® (adalimumab-aaty)	Celltrion	May 2023	UC and CD	CD
Ustekinumab (Stelara®)	Wezlana® (ustekinumab-auub)	Amgen	October 2023	UC and CD	None

## Challenges and risks

The processes involved in developing and producing biologics are extremely intricate, dependent on several factors, and specifically tailored to a single product. Even slight manufacturing alterations can result in considerable variances in the cellular systems employed for biologic development and differences in the finished product's biology, stability, and structure. Unfortunately, any modification can influence the acceptability and efficacy of the marketed product while raising the risk of undesirable immunological reactions [141].

### Immunogenicity

All biologics and biosimilars possess immunogenic potential and can trigger immunological responses such as infusion reactions, mild hypersensitivity, or cross-reactivity with endogenous molecules. This might lead to the production of anti-drug antibodies (ADAs) and have an impact on the pharmacokinetic (PK) and pharmacodynamic (PD) properties [142, 143]. The production of ADAs can lead to a reduction in mAbs PD activity or bioavailability [144]. Moreover, ADAs may result in loss of treatment response or patient non-response [145]. ADAs act as neutralizing antibodies that block mAb active sites and cancel their effect or engage different mAb domains that may speed up mAb clearance without compromising antigen binding capacity [146]. In the latter situation, circulating ADAs might reduce the number of bioavailable mAbs in the blood, sub-therapeutic level, limiting their potential effect [147–149].

Certainly, several factors contribute to immunogenicity, but they are still not entirely known. According to current opinions of researchers, prediction tools such as therapeutic drug monitoring (TDM), specifically after completion of induction dose, and learning from the clinical impacts of other mAbs previously delivered to patients can all help enhance the evaluation of undesired immunogenicity [142].

To maximize the effectiveness of these biologics or biosimilars, it's crucial to target appropriate patients and optimize their delivery method. At the same time, it's crucial to implement preventive approaches before and throughout therapy to reduce the risk of side effects. Subsequently, the benefit-to-risk ratio significantly favors the benefits of biologics or biosimilars by minimizing side effects and maximizing their effectiveness [150]. Although biologics and biosimilars are widely used, safety issues are the main challenges up till now [151]. The most common safety issues include raised risk of infections, hypersensitivity, autoimmunity, development of malignancies, liver toxicity as well as worsening of heart failure.

###  The risk of infection

Linked to the use of biologics and biosimilars can be conceptualized in terms of bacterial infections, viral infections (e.g. hepatitis B), fungal infections, uncommon but potentially fatal opportunistic infections, and reactivation of latent TB [152, 153].

### Hypersensitivity

To biologics and biosimilars can be immediate or delayed reactions. Immediate reactions appear within 30–120 min of administration and may result from infusion-related reactions and type I hypersensitivity reactions that lead to serious systemic reactions, including anaphylaxis [154–156]. On the other hand, delayed responses include type III (serum sickness reactions) and type IV reactions, which may involve rash, fever, pruritis, purpura, malaise, myalgia, arthralgia, and edema [155, 156].

### Autoimmunity

Biologics and biosimilars, particularly anti-TNF-α, might cause T-cell apoptosis and DNA release, potentially leading to the production of autoantibodies against double-stranded DNA (anti-dsDNA) and antinuclear antibodies (ANAs). This may increase the risk of lupus-like syndrome and future development of autoimmune diseases [157, 158].

### The risk of developing malignancies

Is an additional concern for patients receiving biologics or biosimilars. Malignancies, including lymphomas, have been reported among children, adolescents, and young adults who received anti-TNF-α [114]. US-FDA-reported risks and challenges related to biosimilars and their reference biologics are stated in Table 3.

**Table 3:** US-FDA-reported risks and challenges related to biosimilars and their reference biologics

Biologics/Biosimilars	US-FDA-reported risks and challenges	Ref
Infliximab	Serious infections, malignancies, hepatitis b virus reactivation, heart failure, hepatotoxicity, hematologic reactions, hypersensitivity, autoimmunity, reactivation of latent TB, and cardiovascular and cerebrovascular reactions	[159]
Infliximab-dyyb	As its reference (infliximab)	[160]
Infliximab-abda	As its reference (infliximab)	[161]
Infliximab-qbtx	As its reference (infliximab)	[162]
Infliximab-axxq	As its reference (infliximab)	[163]
Adalimumab	Serious infections, malignancies, hepatitis b virus reactivation, hypersensitivity, autoimmunity, cytopenias, pancytopenia, liver enzyme elevation, heart failure, reactivation of latent TB, and rarely neurological disorders like demyelinating disease and optic neuritis	[138]
Adalimumab -atto	As its reference (adalimumab)	[164]
Adalimumab-adbm	As its reference (adalimumab)	[165]
Adalimumab-adaz	As its reference (adalimumab)	[166]
Adalimumab-bwwd	As its reference (adalimumab)	[167]
Adalimumab-afzb	As its reference (adalimumab)	[168]
Adalimumab-fkjp	As its reference (adalimumab)	[169]
Adalimumab-aqvh	As its reference (adalimumab)	[170]
Adalimumab-aacf	As its reference (adalimumab)	[170]
Adalimumab-aaty	As its reference (adalimumab)	[172]
Ustekinumab	Infections, malignancies, hypersensitivity, posterior Reversible Encephalopathy Syndrome (PRES), reactivation of latent TB, noninfectious pneumonia, and vulnerability to particular infections such as mycobacteria and salmonella in IL12/23 deficient patients	[171]
Ustekinumab-auub	As its reference (ustekinumab)	[172]

Several approaches and strategies should be taken into consideration to minimize the aforementioned risks. Pre-treatment measures include obtaining a complete medical history from patients, doing a physical examination, and ruling out latent TB infection and sepsis. Vaccinating patients against vaccine-preventable illnesses (such as influenza) is also suggested. Throughout their course of therapy, patients must be regularly monitored, and any complaints that arise should be addressed promptly [150].

## Delivery systems used for biologics and biosimilars for the treatment of IBD

The serious and alarming systemic side effects discussed above, in addition to those caused by IV and SC injections, have motivated researchers to seek innovative DDSs [173]. The primary objective was to mitigate these adverse effects while simultaneously enhancing therapeutic outcomes. By employing advanced drug delivery technologies, such as microparticles, nanoparticles, targeted delivery systems, stimuli-responsive DDSs, and controlled-release mechanisms, researchers aimed to minimize systemic exposure to the drug while maximizing its efficacy at the site of inflammation. These innovative approaches hold the potential to revolutionize the treatment landscape for IBD by offering safer and more effective therapeutic options for patients. Oral drug delivery for the management of IBD is appealing from a therapeutic point of view due to direct drug delivery to the disease site, resulting in improved efficacy and reduced side effects. Patient-wise, it is also preferred due to the difficulties and side effects associated with parenteral administration [174]. The literature shows several strategies for drug delivery to the inflamed sites in GIT. These include conventional techniques such as time-dependent drug release in the colon, prodrugs, osmotic-controlled and pressure-controlled systems, and others which were reviewed in a previous publication [175]. More recently, microparticle- and nanoparticle-based DDSs have emerged as new tools to facilitate drug targeting to the inflamed sites in the GIT [176, 177]. Microparticle- and nanoparticle-based drug delivery strategies are dependent on their size and charge, change in the pH or pressure in the GIT, polymer degradation, nanoparticle decoration with specific ligands, and microbiome-dependent drug release [177–180].

### Passive targeting strategies

**Fig. 3:**
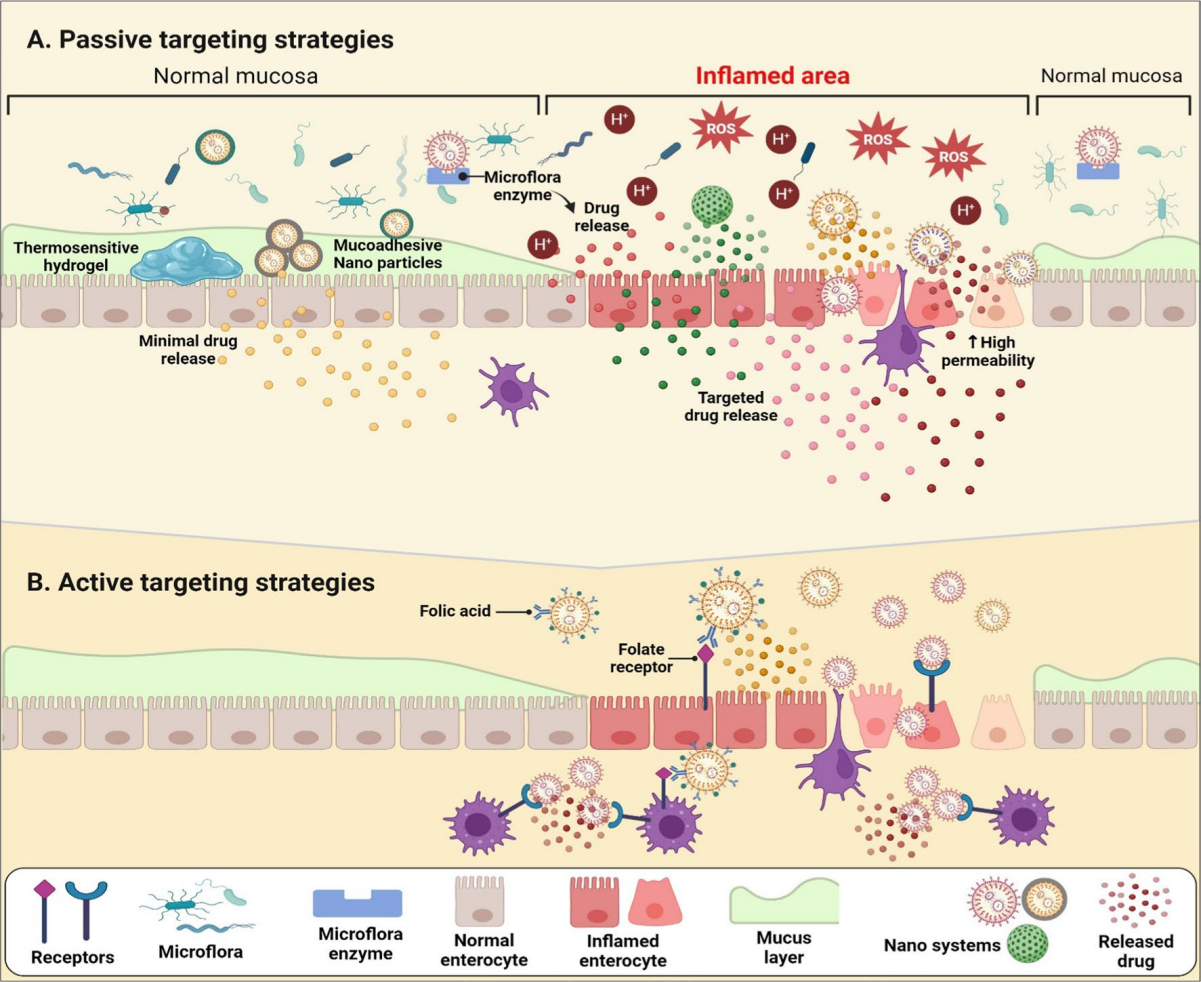
Different approaches for targeted drug delivery systems for IBD management. **A** passive targeting strategies depend on unique pathophysiological alterations observed in inflamed intestinal tissue to deliver drugs specifically to the affected sites, such as increased permeability, heightened concentrations of reactive oxygen species (ROS), disease-induced pH fluctuations, variations in mucus production, and elevated levels of enzymes. **B** Active targeting strategies depend on specific interactions with receptors that exhibit elevated concentrations in IBD-affected tissues, such as CD44, CD98, and folate receptors. Created with BioRender

Passive targeting of DDSs for the management of IBD typically takes advantage of the unique pathophysiological alterations observed in inflamed intestinal tissue to deliver drugs specifically to the affected sites. Features such as increased vascular permeability, heightened concentrations of reactive oxygen species (ROS), disease-induced pH fluctuations, variations in mucus production, elevated levels of enzymes and receptors, and inflammatory cell infiltration are exploited to deliver therapeutic drug concentrations to the disease's sites [181] (Fig. 3). While many of these approaches have been extensively described and investigated for conventional IBD drugs, their applicability to biologics and biosimilars remains largely unexplored. This represents a promising area warranting further research. The following sections will shed light on the most commonly studied passive targeting approaches in the management of IBD.

#### Enhanced permeability and retention effect

The attractive features of nanoparticles include their small size, which facilitates permeation through biological membranes. This is advantageous in the case of CD due to its transmural nature [182]. In the inflamed colon, the permeability of intestinal tissue is heightened, affecting both the endothelium and the epithelium (Fig. 3). This is somehow similar to the enhanced permeability observed in tumor tissues and could, therefore be exploited to augment drug delivery in the inflamed colon [183, 184].

#### Mucoadhesive DDSs

Mucoadhesive nanoparticles and delivery systems are another approach to increasing drug residence time and efficacy in the inflamed GIT areas [185, 186] (Fig. 3). Previous research showed that the mucoadhesion strength was affected by the nanoparticle size. For instance, fluorescent polystyrene particles of sizes 10 mm, 1 mm, and 100 nm were administered by oral gavage to healthy and trinitrobenzene sulfonic acid (TNBS)-induced colitic rats [187]. Higher adherence of particles was observed in the thicker mucus layer and the ulcerated regions. The highest deposition of the particles was observed for the 100-nm nanoparticles. Although several other studies showed increased nanoparticle adhesion to the inflamed tissue in the colon compared to microparticles in experimental animal models, the opposite effect was observed in human IBD patients [183]. Thus, Schmidt et al. compared the uptake of fluorescently labeled placebo nanoparticles with a size of 250 nm and microparticles with a size of 3.0 μm in IBD human patients. The microparticles showed significantly enhanced accumulation in ulcerous lesions of IBD patients compared with healthy control subjects. The nanoparticles showed only small adherence on the mucosal surfaces of all patients [188]. Further research is needed in this area to clarify the reasons for this discrepancy.

The practice of coating nanoparticles with poly ethylene glycol (PEG), commonly known as PEGylation, represents a widespread strategy aimed at enhancing the stability of nanoparticles within the GIT. This approach also concurrently enhances their absorption potential. In a study aimed to develop a mucus-penetrating DDS, poly lactic-co-glycolic acid (PLGA) nanoparticles with two different PEG chain lengths (2 kDa and 5kDa) were used to encapsulate an anti- TNF-α antibody for the management of IBD [189]. Both nanoparticle preparations effectively reduced TNF-α secretion from lipopolysaccharide-stimulated macrophages and intestinal epithelial cells (caco-2 cell line). Despite similar in vitro efficacy, only the nanoparticle coated with PEG 2 kDa had better colon weight/length ratio, histological score, and reduced tissue-associated myeloperoxidase activity and expression of TNF-α levels compared with the control group in an experimentally-induced acute colitis model in mice. In another study, PEG- and chitosan-coated PLGA nanoparticles were tested for their deposition and permeability through healthy and inflamed intestinal mucosa using macrobiopsies from healthy and IBD patients and compared with uncoated nanoparticles. Chitosan-PLGA nanoparticles had the lowest particle permeability and deposition in healthy and inflamed tissues. In contrast, PEG-PLGA nanoparticles showed a more than twofold increase in nanoparticle translocation through inflamed mucosa compared with the healthy mucosa. The translocation of PEG-PLGA microparticles was lower than that of the nanoparticles. The deposition in the inflamed tissue (10.8%) of PEG-PLGA microparticles was much higher than that in healthy tissue (4.1%) [190]. Surface functionalization also affected the in vitro and in vivo behavior of mesoporous silica nanoparticles coated with either PEG or hyaluronic acid (HA) [191]. Thus, PEG-coated nanoparticles accumulated in the lower intestinal tract of healthy animals, whereas HA-coated nanoparticles had better accumulation in the inflamed tissue. Inflammation promoted retention of HA-functionalized particles in a dextran sulfate sodium (DSS)-induced colitis model. Systemic absorption of both nanoparticle-systems was low.

#### Targeting based on surface charge

The presence of negatively charged colonic mucin, attributed to its negatively charged carbohydrate moieties, is another characteristic of IBD that can be exploited in the development of targeted DDSs. These systems have the potential to enhance drug residence time in the colon by facilitating surface binding through electrostatic interactions [192]. Chitosan is one of the widely studied naturally occurring cationic polymers due to its biocompatibility, safety, and biodegradability. Several studies showed its potential for the preparation of cationic DDSs for site-specific drug delivery to the inflamed colon [193–197].

#### Thermosensitive DDSs

Thermosensitive DDSs offer promising avenues for the treatment of IBD due to their response to temperature changes, typically changing from solution to gel at physiological temperatures. This property allows them to form a gel-like depot upon administration, which can prolong drug release and enhance local drug concentrations at the inflamed IBD tissue [198].

A topical thermosensitive mucoadhesive hydrogel system made from chitosan, poloxamer 188, and poloxamer 407 and containing PEG-poly(ε-caprolactone) (PEG-PCL) micelles was used for the rectal delivery of cyclosporine A in a TNBS-induced colitis model in mice [199]. Following rectal administration rapid gelation and prolonged retention on the inflamed colon were noticed. In addition, the hydrogel regulated the expression levels of proinflammatory cytokines (TNF-α, IL-1β, COX-2, and iNOS2) and anti-inflammatory cytokines (IL-10, Nrf2, NQO1, and HO-1).

#### Microflora-based drug targeting

The colon contains a diverse microbiota, comprising a multitude of bacterial species that play pivotal roles in gut health. Within this ecosystem, bacteria produce a vast array of enzymes that can degrade various polysaccharides. DDSs coated with polysaccharides are highly appealing for colon-targeted drug delivery. This is attributed to their resistance to dissolution in the upper GIT and their specific digestion by the colonic microflora [200]. In this context, we have prepared pectin/sodium carboxymethylcellulose microspheres based on the specific degradation of pectin in the colon by the pectinase enzyme and the slow pH-dependent swelling of sodium carboxymethylcellulose [201]. The microspheres exhibited pH-dependent swelling, limited drug release in simulated gastric fluid, and sustained-release in simulated small intestinal fluid. Additionally, they underwent accelerated degradation in the presence of rat caecal contents.

#### pH-triggered drug targeting

Inflammation in the colon can lead to considerable fluctuations in pH levels, with research indicating that colonic pH tends to be notably more acidic in individuals with IBD. Studies have revealed that pH levels in the colons of IBD patients can range from 2.3 to 5.5, contrasting with the typical pH of 7.0 ± 0.7 observed under normal conditions [202]. In this context, we have prepared chitosan beads loaded with either azathioprine (AZA) or quercetin (QUR) and filled them into acid-resistant capsules to avoid premature drug release in the stomach [178, 179]. The results showed that the highest drug release was observed in IBD-simulating pH conditions. Drug-loaded beads were evaluated in a rabbit model of acetic acid-induced colitis where they showed enhanced therapeutic efficacy compared with untreated controls and free drugs. The literature shows several other review articles addressing the utilization of this approach and others for the specific delivery of non-biological treatments for IBD colon [203–206].

### Active targeting strategies

The surface of micro- or nanoparticles can be strategically modified to incorporate specific moieties that respond to pathophysiological changes unique to IBD or to interact with receptors that exhibit elevated concentrations in IBD-affected tissues. Through these modifications, the particles can be designed to achieve localized and targeted drug delivery to the inflamed sites associated with IBD. This approach capitalizes on the disease's distinctive biochemical milieu or receptor expression patterns, facilitating enhanced therapeutic efficacy while minimizing systemic side effects. For instance, CD44, CD98, and folate receptors are overexpressed in inflammatory tissues (Fig. 3). The presence of high concentrations of CD44 in pro-inflammatory macrophages was exploited to prepare hyaluronic acid-bilirubin (HA-BN) nanoparticles that accumulate specifically in the inflamed colon through interactions between HA and CD44 receptors [207]. The nanoparticles accumulated in inflamed colonic epithelium and restored the epithelium barriers in DSS-induced acute colitis in female C57-BL/6 mice. The nanoparticles additionally influenced the gut microbiota, leading to an increase in overall richness and diversity, while notably enhancing the abundance of *Akkermansia muciniphila* and *Clostridium* XIVα.

Another feature of IBD is the high concentrations of ROS due to inflammation-induced oxidative stress. Immune cells in the gut mucosa, such as macrophages and neutrophils, release ROS as a part of the inflammatory response to combat pathogens. However, excessive ROS production can damage tissues and exacerbate inflammation in the intestine [208]. The above-mentioned HA-BN nanoparticles had significant ROS-scavenging activity and protected human HT-colonic epithelial cells from ROS-induced cytotoxicity [207]. The nanoparticles had higher accumulation in the inflamed mice colon and were associated with F4/80 + macrophages in the colonic mucosa compared with the healthy mice control due to the specific interactions of HA with the CD44 receptors. The potential of these nanoparticles as delivery systems for biologics and biosimilars is yet to be tested.

Cellulolytic bacteria exhibit a notably greater prevalence in the colon as opposed to the small intestine [209]. This distinction was used to fabricate solid lipid nanoparticles coated with cellulase-responsive layers and containing budesonide for the treatment of IBD [210].

Folate receptors are overexpressed on a variety of cells, such as cancer cells and activated macrophages, showing that they could be used for the selective delivery of drugs for the treatment of IBD or other diseases [211]. In this regard, ginger active compound, 6-shogaol was loaded into PLGA/PLA-PEG-folic acid nanoparticles, and its therapeutic potential against IBD was tested in a DSS-induced mouse colitis model [212]. The nanoparticles had high folate receptor-mediated uptake by colon-26 cells and activated Raw 264.7 macrophage cells. Oral administration of the nanoparticles significantly improved the colitis symptoms as indicated by reduced levels of several pro-inflammatory mediators.

The combination of different targeting mechanisms increases the possibility of success for IBD-targeted DDSs due to the possibility of loss of selectivity as a result of the complex in vivo environment of the normal and inflamed GIT [177]. For instance, an enzyme- and pH-responsive hydrogel was prepared from 2-hydroxyethyl methacrylate and methacrylic acid copolymer and cross-linked with an azobenzyl cross-linker [213]. Metronidazole and mesalamine were employed as model drugs for anti-IBD applications. The hydrogel demonstrated pH-dependent swelling behavior, with the lowest degree of swelling observed at pH 1.2 and the highest at pH 7.4. Consequently, drug release was attenuated in gastric and small intestine environments, while it was augmented in colonic media containing cecal matter. Another dual-targeting system was prepared from ROS-responsive, folic acid-functionalized nanoparticles [214]. The nanoparticles were able to protect cells from H_2_O_2_-induced oxidative damage. The nanoparticles were also internalized by RAW 264.7 and colon-26 cells effectively and were localized to the inflamed colon. When tested in vivo in a murine DSS-induced colitis model, the nanoparticles showed promising anti-inflammatory activities due to dendritic cell regulation, macrophage polarization promotion, and T-cell infiltration regulation.

Most of the strategies mentioned above were primarily utilized for the delivery of non-biologics, and their applicability could, therefore be extrapolated to biologics and biosimilars. The subsequent sections will elucidate the utilization of these diverse strategies as delivery systems for biologics and biosimilars in managing IBD, with a particular emphasis on infliximab and adalimumab due to their widespread use as TNF-α blockers.

### Polymeric micro- and nanoparticles for the delivery of biologics

One challenge facing the delivery of biologics such as infliximab and adalimumab is the extensive range of side effects associated with the long-term systemic administration, as well as those difficulties facing oral drug delivery. For instance, oral administration of biologics is confronted with the harsh enzymatic and pH environment of the GIT, resulting in a loss of biological activity due to the protein nature of these biologics [215]. In addition, the large molecular weight and ionic nature of biologics limit their permeability through the intestinal epithelium [216]. To overcome the barriers associated with oral administration, several approaches and delivery systems are under active investigation, such as chemical modification and encapsulation into hydrogels, microparticles, or nanoparticles [217, 218].

A hybrid nanopolyplexe system consisting of anionic infliximab, anionic carboxymethyl chitosan, and cationic chitosan was developed and loaded in calcium alginate microparticles [219]. The preparation was evaluated in a TNBS-induced colitis model in female C57BL/6 mice. The results showed that the developed nano-in-microparticles system mitigated inflammation, maintained the intestinal epithelial barrier, and reduced systemic exposure to infliximab. In another study, infliximab was loaded into polymeric PLGA-PEG nanoparticles and tested in a healthy and inflamed caco-2 cell line model for the intestinal epithelial barrier [220]. Infliximab was loaded into the nanoparticles through adsorption via electrostatic interactions to circumvent the deleterious effects on its stability encountered during conventional nanoparticle preparation. The results showed that the nanoparticles had significantly enhanced interaction with the inflamed cells compared with the healthy ones. Cumulative nanoparticle transport through healthy and inflamed cells was affected by particle size and polydispersity. Nanoparticle transport across the inflamed cells was significantly higher than the healthy ones. This effect was ascribed to the inflammatory cytokines-mediated increase in barrier permeability through actin restructuring and endocytosis of tight junction proteins [221]. In another study, infliximab was loaded into self-assembled supramolecular nanoparticles made from tannic acid and PEG [222]. When administered orally to a DSS-induced colitis model in female C57BL/6 mice the nanoparticles increased infliximab accumulation in the inflamed colon compared with free drug. Several measurable parameters, such as average colon weight, length, and inflammatory factors in serum and colon after treatment with infliximab-loaded nanoparticles were comparable to those in healthy control.

To reduce systemic adalimumab exposure, reduce its systemic side effects, and improve its stability against proteolytic degradation, it was covalently linked to the PLGA nanoparticle surface [223]. Results showed that the nanoparticles had a strong stabilization effect on adalimumab against proteolytic degradation by papain. Moreover, intra-colonic delivery of adalimumab nanoparticles demonstrated a more efficient reduction in the levels of pro-inflammatory mediators such as TNF-α, MPO, and IL-1β compared to the drug solution. This led to a decreased severity of clinical symptoms associated with IBD in a murine experimental colitis model. The intracolonic administration also reduced the systemic drug exposure compared with subcutaneous or rectal administration which potentially could lead to better systemic side effects profile.

## Challenges regarding DDSs

Microparticles and nanoparticles present a significant potential for revolutionizing the management of inflammatory bowel disease (IBD) by enabling disease-specific drug release. These innovative DDSs may improve drug efficacy, minimize side effects, and enhance overall patient outcomes. However, the translation of these approaches from laboratory studies to clinical practice faces several significant challenges that must be addressed.

Among these, nanoparticle preparation and characterization present critical obstacles. Achieving uniform particle size, shape, drug loading capacity, release rate, and surface characteristics is often difficult and can vary significantly between batches [177, 203]. The stability of DDSs and encapsulated drugs, both in vitro and in vivo, is vital to maintaining their efficacy and safety. Furthermore, ensuring reproducibility in manufacturing processes is crucial for the scalability and widespread clinical adoption of these systems [217].

In terms of clinical applicability, while the promising potential of DDSs is recognized, their implementation in real-world clinical practice will require substantial preclinical and clinical research to prove their safety, efficacy, and regulatory compliance. Extensive studies are required to demonstrate the quality standards of DDSs and to gain acceptance from regulatory agencies [224]. These preclinical and clinical trials will be crucial to translating laboratory findings into clinically viable solutions.

## Conclusion and future perspectives

Biosimilars are gaining more attention as alternatives to biologics to manage patients with UC and/or CD. Studies have demonstrated that biosimilars offer efficacy and safety profiles comparable to their reference biologics and may improve patient compliance due to their reduced cost. Currently, 14 biosimilars have been approved for IBD management, with more expected in the future. However, post-marketing surveillance has revealed certain risks associated with these treatments, including increased risk of infections, hypersensitivity, autoimmunity, malignancies, liver toxicity, and worsening heart failure. These concerns should be carefully considered in future research to minimize potential health risks. Despite the promising outcomes of biologics and biosimilars, several challenges limit their clinical effectiveness. One of the key barriers is the large molecular size of these biologics, which often requires specialized DDSs to enhance stability, bioavailability, and efficacy.

However, significant preclinical research is still needed before DDSs can be widely implemented in clinical practice. Future research endeavors in this field should focus on advancing the delivery of biologics and biosimilars through nanoparticle-based systems. Biologics and biosimilars, due to their large molecular size, complex structure, and ionic nature, present formidable challenges in oral delivery. Research should prioritize innovative solutions that address issues such as protection from enzymatic degradation, enhancement of mucosal penetration, improvement of site-specific drug delivery, and minimizing systemic drug exposure. Addressing these challenges holds the key to unlocking the full therapeutic potential of nanoparticle-based DDSs in the management of IBD and other complex diseases.

## Data Availability

No datasets were generated or analysed during the current study.
